# Robust competitive facility location model with uncertain demand types

**DOI:** 10.1371/journal.pone.0273123

**Published:** 2022-08-17

**Authors:** Wuyang Yu

**Affiliations:** School of Management, Hangzhou Dianzi University, Hangzhou, Zhejiang Province, China; Sant Longowal Institute of Engineering and Technology, INDIA

## Abstract

In competitive settings, firms locate their facilities according to customers’ behavior to maximize their market share. A common behavior is consuming from different motivations: one is for convenient demand, and the other is for quality demand. In this behavioral pattern, consumers patronize facilities within convenience for some demands, and patronize high quality facilities beyond convenience range for other demands. This behavior has never been included in competitive facility location problems. Given several other companies’ facilities in the market offering similar products or services, we study how a new entrant company can locate facilities based on this customer behavior to maximize its market share. A two-level robust model for the new entrant company is proposed to locate its facilities by taking into account the uncertainty of the types of customers’ demands. For medium size problems, we propose an equivalent mixed binary linear programming to obtain exact solutions. For large size problems, an exact algorithm (GCKP-A) for solving the inner-level model is given first by exploring the optimal solution. Then a heuristic algorithm is proposed by imbedding (GCKP-A) and 2-opt strategy into the framework of the improved ranking-based algorithm. The performance of the proposed heuristic algorithm is checked for different size problems. The sensitivity analysis of a quasi-real example shows that: (1) in most cases, the uncertainty between two types of demands does not affect the location scheme; (2) the convenience range, the quality range and the quality threshold play an important role in the market share of the new entrant company.

## 1 Introduction

Market competition inevitably exists in most business environments. With proper regulation, market competition can promote econmic develpoment and technological progress, and provide better services to customers. The competitive facility location problem (CFLP) refers to determining the location of new facilities in a competitive market to maximize the available market share [1, 2]. CFLP arises in a wide variety of applications including locating school, preventive health care facilities, chain stores, park-and-ride facilities, charging stations for electric vehicles, etc. Due to the practicality of CFLP in determining the location of commercial facilities, many models have been proposed to describe various attributes of CFLP from different aspects, such as customer choice behavior, strategic space, competitive environment, etc [3].

Customer choice behavior refers to the behavior pattern of customers patronizing different facilities that provide similar products or services. Given facilities that can offer similar products or services, customer behavior plays an important role in estimating the market share that each facility can obtain. The binary rule and the proportional rule are the two most common rules used to describe customer choice behavior in the literature [4]. The binary rule assumes that customers always visit the most attractive facilities, it can be traced back to Hotelling’s linear market duopoly model [5]. The proportional rule was first proposed by Huff [6], which assumes that customers distribute their purchasing power among all facilities in proportion to the attractiveness of the facility. From the perspective of utilities that customers received from facilities, both the binary rule and the proportional rules are forms of behavior determined by utilities. Due to the diverse behaviors of customers in choosing facilities in reality, scholars have proposed a variety of choice rules to describe different customer behaviors.

A common behavior is consuming from different motivations: one is for convenient demand, and the other is for quality demand. In this behavioral pattern, consumers patronize facilities within convenience for some demands, and patronize high quality facilities beyond convenience range for other demands. For example, people usually go to some nearby places for entertainment on weekends, and for some places with higher attractiveness, such as Disneyland, even if the distance is far, people still choose the right time to visit. This phenomenon shows that there are two types of customer demands: one is for convenience and the other is for quality. In addition, through market research, companies can obtain the value range of the proportion of convenience-type (or quality-type) demands in all customer demands, but it is difficult to obtain the specific value of this proportion in each customer. These are the two motivations of this article: (1) Propose a new rule describing this kind of customer behavior; (2) Study the applicability of robust optimization method in dealing with uncertain demand types. To deal with the first motivation, we considered two different radii, the smaller one is for convenience-type demands, and the larger one is for quality-type demands. We use a threshold to distinguish whether a facility is of high quality or not. Aiming at the second motivation, a two-layer robust model is proposed, in which the inner-layer model is used to solve the impact of uncertain demand types on market share. Applying duality theory to the inner-layer model, after proper linearization, the two-layer robust model can be transformed into a mixed integer linear programming. Therefore, small and medium problems can be solved directly by optimization software. For large-scale problems, we first prove a theorem for the optimal solution of the generalized continuous Knapsack problem. Then we propose an exact algorithm (GCKP-A) to solve the inner-layer model. Then a heuristic algorithm is proposed by embedding (GCKP-A) and 2-opt strategy into the framework of the improved ranking-based algorithm. The performance of the proposed heuristic algorithm is checked on 40 benchmark instances. The sensitivity analysis results of a quasi-real example show the impact of different parameters on the location of competitive facilities.

The paper is structured as follows. Section 2 presents a brief literature review of competitive facility location problems. Section 3 proposes a new customer choice rule to describe the customer behavior presented in section 1. Section 4 establishes the robust competitive facility location model. Section 5 gives the exact algorithm for the inner-layer problem, and proposes a heuristic algorithm by embedding the inner algorithm into the framework of the ranking-based procedure. Section 6 reports the computational results comparing the performance of our approach with other approaches in the literature. Finally, Section 7 concludes.

## 2 Literature review

Depending on how competitors respond to each other’s decisions, the main research streams in the competitive facility location problems can be divided into three categories [7]:

(1) Static competition: In this case, a new firm enters a market and provides similar products or services as existing competitors. The basic assumption is the rivals take no action against the newcomer. For example, Drezner et al. [8] extended the proportional rule by assuming randomly distributed facility attractiveness, they proposed two effective methods to solve the competitive facility location problem to analyze “effective” attractiveness. Fernández et al. [9] studied a variant of the proportional rule, assuming that customers only patronize those facilities that they find attractive to be greater than or equal to a threshold. Marianov et al. [10] introduced the customer choice behavior called comparison-shopping in competitive facility location problem. Comparison-shopping means that customers visit multiple stores selling different products, make comparisons before making a purchase decision. By assuming the utility of a customer consists of two parts: a measurable utility value and its non-observable part, Ljubić and Moreno [11] adopted the multinomial logit model to estimate the captured customer demands and proposed a method to solve the model by combining two types of cutting planes. Ahmadi and Ghezavati [12] used two sustainablility measures, flexibility and productivity, to develop an attractiveness function for each facility. They proposed a chance constraint to control the dissatisfactiion in the waiting time for services according to the Jackson Markov network. Mahmoodjanloo et al. [13] studies the multimodal competitive hub location pricing problem for a new company planning to enter a market where an existing competitor operates its hub-and-spoke network. They propose a scatter search algorithm based on a nested approach to solve the model. Mai and Lodi [14] investigated the maximum capture facility location problem by assuming that customers choose facilities according to random utilities. Taking advantage of the convexity and separable structure of the objective function, they proposed an enhanced outer approximation algorithm. Aboolian et al. [15] developed a generalized framework of the competitive facility location and design problem, this generalized facility locatoin and design problem include many classic location models as special cases. Fernández et al. [16] used a Pareto-Huff customer choice rule in the competitive facility location problem, and proposed a heuristic procedure to obtain the best approximate solutions.

(2) Dynamic competition: This type of competition is characterized by the assumption that competitors can respond to new entrants. In general, the strategic decisions of competitors are different to change due to high costs, and usually only the operational strategies of competitors are considered. Dynamic competition is often modeled as a Nash equilibrium problem, which is solved using differential systems. For example, Fahimi et al. [17] considered the competitive supply chain network design problem in which two competitors enter the market at the same time. They derived the equilibrium condition and established the finite-dimensional variational inequality formulation, then proposed an algorithm to solve the problem. Zhou et al. [18] proposed a game-theoretical model to study the price competition between two recyclers of electrical and electronic equipment waste. Cai et al. [19] studied the price and warranty competition between two risk-averse retailers by assuming that the retailers’ risk-aversion levels are private information. Shalouhi et al. [20] studied Nash and Stackelberg models of competition problems in two pharmaceutical supply chains in which both exclusive retailer and manufacturer are included. Sazvar et al. [21] developed a scenario-based multi-objective programming model to design a sustainable closed-loop pharmaceutical supply chain, which simultaneously considers competitive market and demand uncertainties. Yu and Khan [22] employed stochastic programming and fuzzy mathematical programming to establish a multi-objective uncertain equilibrium model for the green supply chain network, and they integrated several different methods to solve the model.

(3) Competition with foresight: The newcomer makes logical decisions by forecasting the possible reactions of the competitor after its entry. Qi et al. [23] considered a variant of the proportional rule that customers only patronize facilities within a range they feel is convenient. They proposed a hybrid tabu search algorithm to solve the corresponding competitive facility location problem. Yu [24] studied the leader-follower competitive facility location problem with the partially proportional rule, this rule assumes that a customer chooses the most attractive firm at first then patronizes all facilities of this firm according to proportional rule. Santos-Peñate et al. [25] considered the competitive facility location problem with the binary and S-shaped customer choice rule, they proposed a matheuristic procedure combining the kernal search algorithm with linear programming for the follower problem. Saha et al. [26] used a multinomial logit model to represent customers’ preferences, and proposed a non-linear integar programming model for the joint facility location and inventory problem with partial-disruption risk. Yu and Khan [27] built an evolutionary game model based on the relationship between agricultural product suppliers and urban residents in the financing system. Lin et al. [28] investigates a bilevel competitive facility location problem to maximize expected reveue by considering a discrete choice rule. Latifi et al. [29] studied the leader-follower competitive facility location problem in a closed-loop supply chain, where customer behavior is the Huff gravity-based rule. By replacing the inner level program with its corresponding Karush-Kuhn-Tucker conditions, they proposed an improved branch-and-refine algorithm to solve this problem.

The above literatures mentioned above combine different customer choice rules to determine the market share a facility can obtain, but none of the rules are suitable for describing the customer behavior described in the introductin of this paper. Therefore, in order to better describe this kind of customer behavior, we need to propose a new customer choice rule, and then study the competitive facility location problem on this basis, so as to provide a useful reference for decision makers.

## 3 New customer choice rule

**Fig 1:**
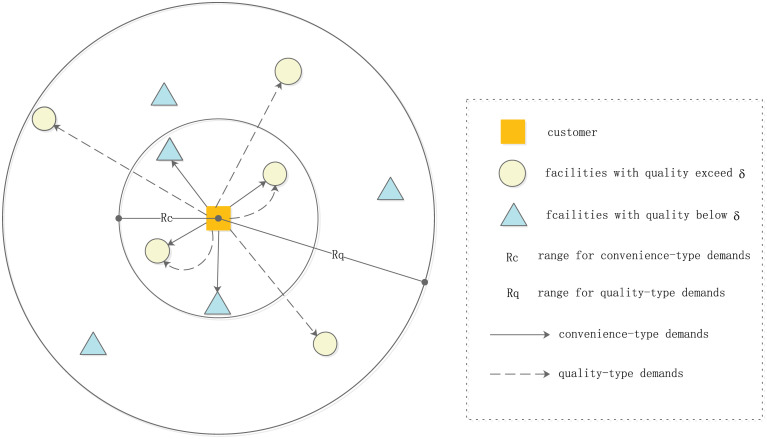
Patronage pattern from the perspective of customer.

In this paper, we assume that every customer has two different types of demands. One is the convenience-type demands, which requires the facilities that meet this type of demand are within the convenience range of customers. The other is the quality-type demands, which requires the facilities that meet this type of demand to have sufficient quality and its range is larger than the convenience range. That is, we consider two patronizing radii, a smaller radius (*R*_*c*_) for convenience-type demands and a larger radius (*R*_*q*_) for quality-type demands. The latter can only be used for facilities whose quality exceeds a given threshold (*δ*). From the perspective of customer, the patronage pattern of customers is shown in Fig 1.

**Fig 2:**
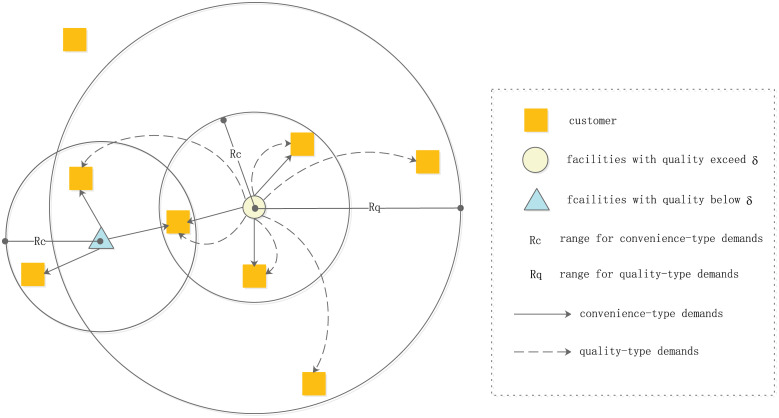
Patronage pattern from the perspective of facility.

It can be seen from Fig 1 that customers only patronize facilities within the range of *R*_*c*_ to meet their convenience-type demands. On the other hand, in order to meet their quality-type demands, customers patronize facilities whose quality exceeds the quality threshold *δ* and falls within the range of *R*_*q*_, where *R*_*q*_ > *R*_*c*_. Note that, no matter the demands are convenience-type or quality-type, the customer behavior follows the proportional rule, in other words, his demands are split by these facilities that satisfy corresponding conditions. If a customer exceeds the *R*_*c*_ range of all facilities, his convenience-type demands cannot be met. Similarly, if a customer exceeds the *R*_*q*_ range of all facilities whose quality exceeds *δ*, his quality-type demands cannot be met. From the perspective of facility, the patronage pattern of customers is shown in Fig 2. It can be seen from Fig 2 that for a facility whose quality exceeds the threshold *δ*, it can meet all types of demands within the range of *R*_*c*_. In addition, it can also meet the quality-type demands between the range of *R*_*c*_ and *R*_*q*_. However, for a facility whose quality is lower than the threshold *δ*, it can only meet the convenience-type demands within the range of *R*_*c*_.

## 4 Robust competitive facility location model

### 4.1 The original two-layer model

An entering company hopes to compete for market share of a region by opening several new facilities, and other companies have already established similar facilities. Assumed that the demands of each customer can be divided into two types: the convenience-type demands and the quality-type demands. The total demands of each customer are fixed and known. Through preliminary market research, companies can obtain the value range of the proportion of convenience-type (or quality-type) demands in all customer demands, but it is difficult to obtain the specific value of this proportion in each customer. Due to a limited budget, the new company needs to determine the location of all new facilities from a set of potential locations in order to obtain the largest market share.

The following notations are used:

*I*: Set of demand points (customers), indexed by *i*;
*J*_*E*_: Set of locations that have established facilities, indexed by *j*;
*J*_*N*_: Set of potential locations for new facilities, indexed by *j*;
*J*: Union of set *J*_*E*_ and set *J*_*N*_, i.e., *J* = *J*_*E*_ ⋃ *J*_*N*_;
*Q*_*j*_: Quality of facility at point *j*, *j* ∈ *J*;
*δ*: The quality threshold of facilities for quality-type demands;
*R*_*c*_: The patronage range of customers for convenience-type demands;
*R*_*q*_: The patronage range of customers for quality-type demands;

$a_{ij}^{C}$ : The attractiveness of facility *j* to the convenience-type demands of customer *i*;

$a_{ij}^{Q}$ : The attractiveness of facility *j* to the quality-type demands of customer *i*;

$w_{i}^{C}$ : The convenience-type demands of customer *i* ∈ *I*;

$w_{i}^{Q}$ : The quality-type demands of customer *i* ∈ *I*;
*w*_*i*_: The total demands of customer *i*, that is, $w_{i}=w_{i}^{C}+w_{i}^{Q},i\in I$;
*W*: The total demands of all customers, $W=\sum_{i\in I}w_{i}$;
*γ*_1_: The lower bound of the proportion of $w_{i}^{C}$ in the total demands *W*, i.e. $0\leq \gamma_{1}\leq \sum_{i\in I}w_{i}/W$;
*γ*_2_: The upper bound of the proportion of $w_{i}^{C}$ in the total demands *W*, i.e. $\sum_{i\in I}w_{i}/W\leq \gamma_{2}\leq 1$;
*f*_*j*_: The fixed cost of establishing a facility in the potential location *j* ∈ *J*_*N*_;
*d*_*ij*_: The distance between the customer *i* and the facility *j*;
*G*: Total budget for the new entering company to build facilities;
*y*_*j*_, *j* ∈ *J*_*N*_: Binary variable, if the potential location *j* is selected to build a facility, then *y*_*j*_ = 1, otherwise *y*_*j*_ = 0.

From the definition of the convenience-type demands and the quality-type demands, we can define the attractiveness of facility *j* to the convenience-type (and quality-type) demands of customer *i* as follows:

$$\begin{array}{c} a_{ij}^{C}=\{\begin{array}{c} \frac{Q_{j}}{1+d_{ij}^{2}},\;\;\text{if}\;\;d_{ij}\leq R_{c}\;\;\;\;\;\;\;\;\;\;\;\;\;\;\;\;\;\;\\ 0,\;\;\;\;\;\;\;\;\;\;\;\text{otherwise} \end{array} \end{array}$$ (1)

$$\begin{array}{c} a_{ij}^{Q}=\{\begin{array}{c} \frac{Q_{j}}{1+d_{ij}^{2}},\;\;\text{if}\;\;d_{ij}\leq R_{q}\;\text{and}\;Q_{j}\geq \delta \\ 0,\;\;\;\;\;\;\;\;\;\;\;\text{otherwise} \end{array} \end{array}$$ (2)

The goal of the new entrant is to maximize market share by setting new facilities in the competitive market under the given budget. Therefore, the competitive facility location problem of the new entering company can be established as the following two-layer programming model:

$$\begin{array}{ccc} & & Z_{R}=\text{max}P\left(y\right)\\ (\text{OM})\;\; & & \;\;s.t.\{\begin{array}{c} \sum_{j\in J_{N}}f_{j}y_{j}\leq G\\ y_{j}\in \left\{0,1\right\},\forall j\in J_{N} \end{array} \end{array}$$

Here the outer-layer model (OM) is used to determine the location of new facilities with a limited budget in order to maximize the market share of the new entrant. According to the previous assumptions, we know that the proportion of $\sum_{i\in I}w_{i}^{C}/W$ is uncertain within the interval [*γ*_1_, *γ*_2_]. Therefore, the following inner-layer model (IM) can be used to deal with the uncertainty of convenience-type demands and quality-type demands of each customer:

$$\begin{array}{ccc} & & P\left(y\right)=\text{min}\sum_{i\in I}[w_{i}x_{i}\frac{\sum_{j\in J_{N}}a_{ij}^{C}y_{j}}{\sum_{j\in J_{E}}a_{ij}^{C}+\sum_{j\in J_{N}}a_{ij}^{C}y_{j}}+w_{i}\left(1-x_{i}\right)\frac{\sum_{j\in J_{N}}a_{ij}^{Q}y_{j}}{\sum_{j\in J_{E}}a_{ij}^{Q}+\sum_{j\in J_{N}}a_{ij}^{Q}y_{j}}]\\ (\text{IM})\;\; & & \;\;\;\;\;\;\;\;\;\;\;\;\;\;s.t.\{\begin{array}{c} \gamma_{1}\leq \sum_{i\in I}w_{i}x_{i}/W\leq \gamma_{2}\\ 0\leq x_{i}\leq 1,i\in I. \end{array} \end{array}$$

Here *x*_*i*_ represents the ratio of convenience-type demands $w_{i}^{C}$ to total demands *w*_*i*_ of customer *i*, i.e. $x_{i}=w_{i}^{C}/w_{i}$. And $\sum_{j\in J_{N}}a_{ij}^{C}y_{j}/(\sum_{j\in J_{E}}a_{ij}^{C}+\sum_{j\in J_{N}}a_{ij}^{C}y_{j})$ represents the convenience-type demands captured by the new entrant from customer *i*.

### 4.2 Linearization of the original model

For brevity, let $h_{i}^{C}\left(y\right):=\frac{\sum_{j\in J_{N}}a_{ij}^{C}y_{j}}{\sum_{j\in J_{E}}a_{ij}^{C}+\sum_{j\in J_{N}}a_{ij}^{C}y_{j}},h_{i}^{Q}\left(y\right):=\frac{\sum_{j\in J_{N}}a_{ij}^{Q}y_{j}}{\sum_{j\in J_{E}}a_{ij}^{Q}+\sum_{j\in J_{N}}a_{ij}^{Q}y_{j}}$, then (IM) is equivalent to:

$$\begin{array}{c} P\left(y\right)=\sum_{i\in I}w_{i}h_{i}^{Q}\left(y\right)+\text{min}\sum_{i\in I}w_{i}x_{i}\left[h_{i}^{C}\left(y\right)-h_{i}^{Q}\left(y\right)\right] \end{array}$$ (3)

$$s.t.\{\begin{array}{cc} \sum_{i\in I}w_{i}x_{i}\geq \gamma_{1}W & \left(4\text{a}\right)\\ -\sum_{i\in I}w_{i}x_{i}\geq -\gamma_{2}W & \left(4\text{b}\right)\\ -x_{i}\geq -1,i\in I & \left(4\text{c}\right)\\ x_{i}\geq 0,i\in I. & \left(4\text{d}\right) \end{array}$$

Suppose the dual variables of constraints (4a–4c) are *u*_1_, *u*_2_, and *v*_*i*_, repectively. Then the dual problem of the minimization part of *P*(*y*) is as follows:

$$\begin{array}{c} \text{max}\;\left(\gamma_{1}W\right)u_{1}-\left(\gamma_{2}W\right)u_{2}-\sum_{i\in I}v_{i} \end{array}$$ (5)

$$s.t.\{\begin{array}{cc} w_{i}\left(u_{1}-u_{2}\right)-v_{i}\leq w_{i}\left(h_{i}^{C}\left(y\right)-h_{i}^{Q}\left(y\right)\right),\forall i\in I & \left(6\text{a}\right)\\ u_{1},u_{2}\geq 0,v_{i}\geq 0,\forall i\in I & \left(6\text{b}\right) \end{array}$$

Therefore, the original two-layer model can be rewritten as a single-layer model as follows:

$$\begin{array}{ccc} & & Z_{R}=\text{max}\sum_{i\in I}w_{i}h_{i}^{Q}\left(y\right)+\left(\gamma_{1}W\right)u_{1}-\left(\gamma_{2}W\right)u_{2}-\sum_{i\in I}v_{i} \end{array}$$ (7)

$$s.t.\{\begin{array}{cc} \sum_{j\in J_{N}}f_{j}y_{j}\leq G & \left(8\text{a}\right)\\ w_{i}\left(u_{1}-u_{2}\right)-v_{i}\leq w_{i}\left(h_{i}^{C}\left(y\right)-h_{i}^{Q}\left(y\right)\right),\forall i\in I & \left(8\text{b}\right)\\ u_{1},u_{2}\geq 0,v_{i}\geq 0,\forall i\in I & \left(8\text{c}\right)\\ y_{j}\in \left\{0,1\right\},\forall j\in J_{N} & \left(8\text{d}\right) \end{array}$$

Since $h_{i}^{C}\left(y\right)$ and $h_{i}^{Q}\left(y\right)$ are nonlinear with respect to *y*_*j*_, the above model is difficult to solve. Therefore, we linearize these two expressions by introducing two new decision variables $\alpha_{i}^{C},\alpha_{i}^{Q}$, let $\alpha_{i}^{C}=h_{i}^{C}\left(y\right),\alpha_{i}^{Q}=h_{i}^{Q}\left(y\right)$.

$$\begin{array}{c} \alpha_{i}^{C}=\frac{\sum_{j\in J_{N}}a_{ij}^{C}y_{j}}{\sum_{j\in J_{E}}a_{ij}^{C}+\sum_{j\in J_{N}}a_{ij}^{C}y_{j}},\;\alpha_{i}^{Q}=\frac{\sum_{j\in J_{N}}a_{ij}^{Q}y_{j}}{\sum_{j\in J_{E}}a_{ij}^{Q}+\sum_{j\in J_{N}}a_{ij}^{Q}y_{j}} \end{array}$$ (9)

From Eq (9), it can be seen that if $\sum_{j\in J_{N}}a_{ij}^{C}y_{j}=0\;\left(\text{or}\;\sum_{j\in J_{N}}a_{ij}^{Q}y_{j}=0\right)$, then $\alpha_{i}^{C}=0\;\left(\text{or}\;\alpha_{i}^{Q}=0\right)$, otherwise there are

$$\begin{array}{c} \left(\sum_{j\in J_{E}}a_{ij}^{C}\right)\alpha_{i}^{C}+\left(\sum_{j\in J_{N}}a_{ij}^{C}y_{j}\right)\alpha_{i}^{C}=\sum_{j\in J_{N}}a_{ij}^{C}y_{j} \end{array}$$ (10)

$$\begin{array}{c} \left(\sum_{j\in J_{E}}a_{ij}^{Q}\right)\alpha_{i}^{Q}+\left(\sum_{j\in J_{N}}a_{ij}^{Q}y_{j}\right)\alpha_{i}^{Q}=\sum_{j\in J_{N}}a_{ij}^{Q}y_{j} \end{array}$$ (11)

Now introduce two other decision variables $\beta_{ij}^{C},\beta_{ij}^{Q},i\in I,j\in J_{N}$, let

$$\begin{array}{c} \beta_{ij}^{C}=\alpha_{i}^{C}y_{j},\;\beta_{ij}^{Q}=\alpha_{i}^{Q}y_{j} \end{array}$$ (12)

Since *y*_*j*_ is a binary variable, if *y*_*j*_ = 0 there are $\beta_{ij}^{C}=0$ and $\beta_{ij}^{Q}=0$, otherwise there are $\beta_{ij}^{C}=\alpha_{i}^{C}$ and $\beta_{ij}^{Q}=\alpha_{i}^{Q}$.

Therefore, the original model can be linearizing as follows:

$$\begin{array}{c} Z_{R}=\text{max}\sum_{i\in I}w_{i}\alpha_{i}^{Q}+\left(\gamma_{1}W\right)u_{1}-\left(\gamma_{2}W\right)u_{2}-\sum_{i\in I}v_{i} \end{array}$$ (13)

$$\left(\text{LM}\right)\;\;s.t.\{\begin{array}{cc} \sum_{j\in J_{N}}f_{j}y_{j}\leq G & \left(14\text{a}\right)\\ w_{i}\left(u_{1}-u_{2}\right)-v_{i}\leq w_{i}\left(\alpha_{i}^{C}-\alpha_{i}^{Q}\right),\forall i\in I & \left(14\text{b}\right)\\ \left(\sum_{j\in J_{E}}a_{ij}^{C}\right)\alpha_{i}^{C}+\sum_{j\in J_{N}}a_{ij}^{C}\beta_{ij}^{C}=\sum_{j\in J_{N}}a_{ij}^{C}y_{j},\forall i\in I & \left(14\text{c}\right)\\ \left(\sum_{j\in J_{E}}a_{ij}^{Q}\right)\alpha_{i}^{Q}+\sum_{j\in J_{N}}a_{ij}^{Q}\beta_{ij}^{Q}=\sum_{j\in J_{N}}a_{ij}^{Q}y_{j},\forall i\in I & \left(14\text{d}\right)\\ \alpha_{i}^{C}\leq M\sum_{j\in J_{N}}a_{ij}^{C}y_{j},\forall i\in I & \left(14\text{e}\right)\\ \alpha_{i}^{Q}\leq M\sum_{j\in J_{N}}a_{ij}^{Q}y_{j},\forall i\in I & \left(14\text{f}\right)\\ 0\leq \alpha_{i}^{C}-\beta_{ij}^{C}\leq \left(1-y_{j}\right),\forall i\in I,j\in J_{N} & \left(14\text{g}\right)\\ 0\leq \alpha_{i}^{Q}-\beta_{ij}^{Q}\leq \left(1-y_{j}\right),\forall i\in I,j\in J_{N} & \left(14\text{h}\right)\\ \beta_{ij}^{C}\leq y_{j},\forall i\in I,j\in J_{N} & \left(14\text{i}\right)\\ \beta_{ij}^{Q}\leq y_{j},\forall i\in I,j\in J_{N} & \left(14\text{j}\right)\\ u_{1},u_{2}\geq 0,v_{i},\alpha_{i}^{C},\alpha_{i}^{Q},\beta_{ij}^{C},\beta_{ij}^{Q}\geq 0,\forall i\in I,j\in J_{N} & \left(14\text{k}\right)\\ y_{j}\in \left\{0,1\right\},\forall j\in J_{N} & \left(14\text{l}\right) \end{array}$$

Here *M* is a sufficiently large positive number. Constraint (14a) indicates that the total cost of all facilities does not exceed the budget. (14b) are the dual constraints on variables *x*_*i*_ in (4d). Constraints (14c–14f) guarantee that Eq (9) holds, and constraints (14g–14j) ensure that Eq (12) is true. Since this linearization model (LM) is a mixed binary linear programming, any commercial optimization software (such as Gurobi, Cplex, etc.) can solve medium-scale problems.

## 5 Ranking-based imbedding algorithm

For large-scale problems, current commercial optimization software cannot solve them well. Therefore, this section designs a ranking-based heuristic algorithm to solve large-scale problems.

Let $p_{i}:=w_{i}\left[h_{i}^{Q}\left(y\right)-h_{i}^{C}\left(y\right)\right]$ and λ_1_ ≔ *γ*_1_*W*, λ_2_ ≔ *γ*_2_*W*, the minimization part of *P*(*y*) is actually a generalized continuous knapsack problem (GCKP).

$$\begin{array}{ccc} & & \text{max}\sum_{i=1}^{n}p_{i}x_{i}\\ (\text{GCKP})\;\; & & s.t.\{\begin{array}{c} \lambda_{1}\leq \sum_{i=1}^{n}w_{i}x_{i}\leq \lambda_{2}\\ 0\leq x_{i}\leq 1,i=1,2,\cdots ,n. \end{array} \end{array}$$

where *w*_*i*_ > 0, *i* = 1, 2, …, *n* and $0\leq \lambda_{1}<\lambda_{2}\leq \sum_{i=1}^{n}w_{i}$. Please note that we call the problem (GCKP) ‘general’ not only it has upper and lower bounds, but also because the profit *p*_*i*_ may be negative. When the item $\sum_{i=1}^{n}w_{i}x_{i}$ has only an upper bound and all profits *p*_*i*_, *i* = 1, 2, …, *n* are non-negative, this problem is a continuous knapsack problem [30].

Reorder the index *i* so that the sorted index *k*_*i*_(*i* = 1, 2, …, *n*) satisfies:

$$\begin{array}{c} \frac{p_{k_{1}}}{w_{k_{1}}}\geq \frac{p_{k_{2}}}{w_{k_{2}}}\geq \cdots \geq \frac{p_{k_{s}}}{w_{k_{s}}}\geq 0>\frac{p_{k_{s+1}}}{w_{k_{s+1}}}\geq \cdots \geq \frac{p_{k_{n}}}{w_{k_{n}}}. \end{array}$$ (15)

The optimal solution of (GCKP) can be determined by the following theorem.

**Theorem**. In addition to the key item *s* defined in (15), the other two key items *s*_1_ and *s*_2_ are defined as follows:

$$\begin{array}{c} s_{1}=\text{max}\{j:\sum_{i=1}^{j}w_{k_{i}}\leq \lambda_{1}\},s_{2}=\text{min}\{j:\sum_{i=1}^{j}w_{k_{i}}>\lambda_{2}\}. \end{array}$$ (16)

Then the optimal solution x* of (GCKP) is

(Case 1) If *s* < *s*_1_, then
  $$\begin{array}{ccc} & & x_{k_{i}}^{*}=\{\begin{array}{c} 1,\;\;\;\;\;\;\;\;\;\;\;\;\;\;\;\;\;\;\;\;\;\;\;\;\;\;\;\;\;\;\;\;\;\;\;\;\;\;\;\;\;\;i=1,2,\cdots ,s_{1}-1\\ \left(\lambda_{1}-\sum_{i=1}^{s_{1}-1}w_{k_{i}}\right)/w_{k_{s_{1}}},\;\;\;\;\;\;\;i=s_{1}\\ 0,\;\;\;\;\;\;\;\;\;\;\;\;\;\;\;\;\;\;\;\;\;\;\;\;\;\;\;\;\;\;\;\;\;\;\;\;\;\;\;\;\;\;i=s_{1}+1,\cdots ,n \end{array} \end{array}$$ (17)
(Case 2) If *s*_1_ ≤ *s* < *s*_2_, then
  $$\begin{array}{ccc} & & x_{k_{i}}^{*}=\{\begin{array}{c} 1,\;\;\;\;\;\;\;\;\;\;\;\;\;\;\;\;\;\;\;\;\;\;\;\;\;\;\;\;\;\;\;\;\;\;\;\;\;\;\;\;\;i=1,2,\cdots ,s\;\;\;\;\\ 0,\;\;\;\;\;\;\;\;\;\;\;\;\;\;\;\;\;\;\;\;\;\;\;\;\;\;\;\;\;\;\;\;\;\;\;\;\;\;\;\;\;i=s+1,\cdots ,n\;\;\;\; \end{array} \end{array}$$ (18)
(Case 3) If *s* ≥ *s*_2_, then
  $$\begin{array}{ccc} & & x_{k_{i}}^{*}=\{\begin{array}{c} 1,\;\;\;\;\;\;\;\;\;\;\;\;\;\;\;\;\;\;\;\;\;\;\;\;\;\;\;\;\;\;\;\;\;\;\;\;\;\;\;\;\;i=1,2,\cdots ,s_{2}-1\\ \left(\lambda_{2}-\sum_{i=1}^{s_{2}-1}w_{k_{i}}\right)/w_{k_{s_{2}}},\;\;\;i=s_{2}\\ 0,\;\;\;\;\;\;\;\;\;\;\;\;\;\;\;\;\;\;\;\;\;\;\;\;\;\;\;\;\;\;\;\;\;\;\;\;\;\;\;\;\;i=s_{2}+1,\cdots ,n \end{array} \end{array}$$ (19)

**Proof:** (Case 1) If *s* < *s*_1_, there is $0>\frac{p_{k_{s_{1}}}}{w_{k_{s_{1}}}}\geq \cdots \geq \frac{p_{k_{n}}}{w_{k_{n}}}$. Then for any feasible solution *x* that satisfies $\lambda_{1}\leq \sum_{i=1}^{n}w_{i}x_{i}\leq \lambda_{2}$ and 0 ≤ *x*_*i*_ ≤ 1, *i* = 1, 2, …, *n*. We have

$$\begin{array}{ccc} & & \sum_{i=1}^{n}p_{k_{i}}x_{k_{i}}=\sum_{i=1}^{s_{1}-1}p_{k_{i}}x_{k_{i}}+\sum_{i=s_{1}}^{n}\frac{p_{k_{i}}}{w_{k_{i}}}\left(w_{k_{i}}x_{k_{i}}\right)\leq \sum_{i=1}^{s_{1}-1}p_{k_{i}}x_{k_{i}}+\frac{p_{k_{s_{1}}}}{w_{k_{s_{1}}}}\sum_{i=s_{1}}^{n}w_{k_{i}}x_{k_{i}}\\ & & \leq \sum_{i=1}^{s_{1}-1}p_{k_{i}}x_{k_{i}}+\frac{p_{k_{s_{1}}}}{w_{k_{s_{1}}}}\left(\lambda_{1}-\sum_{i=1}^{s_{1}-1}w_{k_{i}}x_{k_{i}}\right)=\frac{p_{k_{s_{1}}}}{w_{k_{s_{1}}}}\lambda_{1}+\sum_{i=1}^{s_{1}-1}\left(\frac{p_{k_{i}}}{w_{k_{i}}}-\frac{p_{k_{s_{1}}}}{w_{k_{s_{1}}}}\right)w_{k_{i}}x_{k_{i}}\\ & & \leq \frac{p_{k_{s_{1}}}}{w_{k_{s_{1}}}}\lambda_{1}+\sum_{i=1}^{s_{1}-1}\left(\frac{p_{k_{i}}}{w_{k_{i}}}-\frac{p_{k_{s_{1}}}}{w_{k_{s_{1}}}}\right)w_{k_{i}}=\sum_{i=1}^{s_{1}-1}p_{k_{i}}+\frac{p_{k_{s_{1}}}}{w_{k_{s_{1}}}}\left(\lambda_{1}-\sum_{i=1}^{s_{1}-1}w_{k_{i}}\right)=\sum_{i=1}^{n}p_{k_{i}}x_{k_{i}}^{*} \end{array}$$

The first inequality is due to $\frac{p_{k_{i}}}{w_{k_{i}}}\leq \frac{p_{k_{s_{1}}}}{w_{k_{s_{1}}}},\forall i=s_{1},\ldots ,n$. The second inequality is due to $\frac{p_{k_{s_{1}}}}{w_{k_{s_{1}}}}<0$ and $\lambda_{1}-\sum_{i=1}^{s_{1}-1}w_{k_{i}}x_{k_{i}}\leq \sum_{i=s_{1}}^{n}w_{k_{i}}x_{k_{i}}$. The third inequality is due to $\left(\frac{p_{k_{i}}}{w_{k_{i}}}-\frac{p_{k_{s_{1}}}}{w_{k_{s_{1}}}}\right)w_{k_{i}}>0,\forall i=1,\ldots ,s_{1}-1$ and $x_{k_{i}}\leq 1$. Therefore, x* in (17) is the optimal solution of (GCKP) when *s* < *s*_1_.

(Case 2) If *s*_1_ ≤ *s* < *s*_2_, the result is obvious.

(Case 3) It can be proved in a similar way to Case 1.

This theorem is proved by us and is not quoted from any other papers, it is a generalization of the optimal solution theorem for the continuous knapsack problem in [30]. The form of this optimal solution is not very intuitive, but the result obtained after combining $w_{k_{i}}$ becomes very intuitive.

$$\begin{array}{ccc} & & \sum_{i=1}^{n}w_{k_{i}}x_{k_{i}}^{*}=\{\begin{array}{c} \lambda_{1},\;\;\;\;\;\;\;\;\;\;\;\;\;\;\;\;\sum_{i=1}^{s}w_{k_{i}}<\lambda_{1}\\ \sum_{i=1}^{s}w_{k_{i}},\;\;\;\;\;\;\;\;\;\;\lambda_{1}\leq \sum_{i=1}^{s}w_{k_{i}}\leq \lambda_{2}\\ \lambda_{2},\;\;\;\;\;\;\;\;\;\;\;\;\;\;\;\;\sum_{i=1}^{s}w_{k_{i}}>\lambda_{2} \end{array} \end{array}$$ (20)

According to the result of Eq (20) and the non-increment of $x_{k_{i}}$, we propose the iterative algorithm for solving the (GCKP) as follows:

**GCKP-A:**
  Step 1: Let $J_{1}≔\left\{j:p_{j}>=0\right\},J_{2}≔\left\{j:p_{j}<0\right\},\bar{W}≔\sum_{j\in J_{1}}w_{j}$. Set $x_{j}^{*}≔1$ for *j* ∈ *J*_1_ and $x_{j}^{*}≔0$ for *j* ∈ *J*_2_.
Step 2: While $\bar{W}<\lambda_{1}$:
    Find the index *j*′ such that $\frac{p_{j^{\prime }}}{w_{j^{\prime }}}=\text{max}\{\frac{p_{j}}{w_{j}}:j\in J_{2}\}$.
    If $w_{j^{\prime }}\leq \lambda_{1}-\bar{W}$, then set $x_{j^{\prime }}^{*}≔1,\bar{W}≔\bar{W}+w_{j^{\prime }}$. Else set $x_{j^{\prime }}^{*}≔\left(\lambda_{1}-\bar{W}\right)/w_{j^{\prime }},\bar{W}≔\lambda_{1}$.
    Update *J*_2_ ≔ *J*_2_\{*j*′}.
   End while.
Step 3: While $\bar{W}>\lambda_{2}$:
    Find the index *j*′ such that $\frac{p_{j^{\prime }}}{w_{j^{\prime }}}=\text{min}\{\frac{p_{j}}{w_{j}}:j\in J_{1}\}$.
    If $w_{j^{\prime }}\leq \bar{W}-\lambda_{2}$, then set $x_{j^{\prime }}^{*}≔0,\bar{W}≔\bar{W}-w_{j^{\prime }}$. Else set $x_{j^{\prime }}^{*}≔1-\left(\bar{W}-\lambda_{2}\right)/w_{j^{\prime }},\bar{W}≔\lambda_{2}$.
    Update *J*_1_ ≔ *J*_1_\{*j*′}.
   End while.
Step 4: Output the optimal solution x* and the objective value $\sum_{j=1}^{n}p_{j}x_{j}^{*}$.

**Fig 3:**
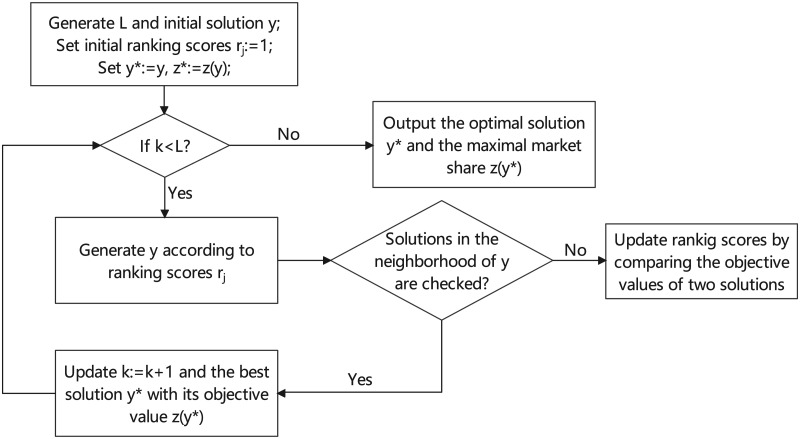
Flowchart of the algorithm (REA).

We know that the inner-layer model (IM) can be accurately solved by (GCKP-A) for any given location scheme of the new entering firm, so the remaining problem is to efficiently solve the outer-layer model (OM). Fernández et al. [16] proposed a new rule: Pareto-Huff customer choice rule, and used the ranking-based heuristic strategy (RDOA) to solve the corresponding competitive facility location problem. By setting a different rank rule, we [31] proposed an improved ranking-based algorithm for the competitive facility location problem. Compared with other heuristic algorithms (such as genetic algorithm, simulated annealing algorithm, ant colony algorithm, etc.), the ranking-based heuristic algorithm performs very well on the facility location problem. Therefore, by embedding (GCKP-A) and the 2-opt strategy, we proposed a ranking-based embedding algorithm (REA) to solve the model of this paper. The flowchart of the algorithm (REA) is shown in Fig 3.

**Ranking-based Embedding Algorithm (REA)**

Step 1. (Initialization of the algorithm):
  Input parameters of the model, set the iterate number *L* and set initial ranking scores *r*_*j*_ ≔ 1, ∀*j* ∈ *J*_*N*_, set λ_*C*_ ≔ (λ_1_+ λ_2_)/2. Randomly choose y* such that $\sum_{j\in J_{N}}f_{j}y_{j}^{*}\leq G$, calculate *P*(y*) by (GCKP-A). Let *k* ≔ 0 and set $Z_{R}^{*}≔P\left(y^{*}\right)$.
Step 2. (Ranking-based algorithm embedding (GCKP-A)):
   While *k* < *L*:
    Step 2.1 (Generate a solution *y* according to the ranking-based rule)
     Let *y*_*j*_ ≔ 0, ∀*j* ∈ *J*_*N*_, calculate the probability *p*_*j*_ of each candidate location *j* ∈ *J*_*N*_:
  $$\begin{array}{c} p_{j}≔\frac{A_{j}r_{j}}{\sum_{j\in J_{N}}A_{j}r_{j}},\forall j\in J_{N},\text{where}A_{j}=\lambda_{C}\sum_{i\in I}a_{ij}^{C}+\left(1-\lambda_{C}\right)\sum_{i\in I}a_{ij}^{Q} \end{array}$$
     Repeat the roulette-wheel selection process to set *y*_*j*_ ≔ 1 until $\sum_{j\in J_{N}}f_{j}y_{j}>G$.
    Step 2.2 (Use 2-opt strategy to update the ranking scores)
     Randomly choose *j*′ ∈ *Y*^1^ ≔ {*j* ∈ *J*_*N*_|*y*_*j*_ = 1}. Let $F^{\prime }≔\{k\in \left(J_{N}\backslash Y^{1}\right)|f_{k}\leq G-\sum_{j\in J_{N}}f_{j}y_{j}+f_{j^{\prime }}\}$.
     For each $\bar{j}\in F^{\prime }$:
      Let $\tilde{y}≔y$. Set ${\tilde{y}}_{j^{\prime }}≔0,{\tilde{y}}_{\bar{j}}≔1$, calculate *P*(*y*) and $P(\tilde{y})$ by (GCKP-A).
      If $P\left(y\right)>P\left(\tilde{y}\right)$:
       Set $r_{j}≔r_{j}+y_{j}\cdot 2,r_{j}≔r_{j}-{\tilde{y}}_{j}$; Update $y^{*}≔y,Z_{R}^{*}≔P\left(y\right)$ when $P\left(y\right)>Z_{R}^{*}$;
      Else:
       Set $r_{j}≔r_{j}+{\tilde{y}}_{j}\cdot 2,r_{j}≔r_{j}-y_{j}$; Update $y^{*}≔\tilde{y},Z_{R}^{*}≔P\left(\tilde{y}\right)$ when $P\left(\tilde{y}\right)>Z_{R}^{*}$.
      Update *r*_*j*_ ≔ *r*_*j*_ + 1, ∀*j* ∈ *J*_*N*_ if any *r*_*j*_ = 0.
     End for.
    *k* ≔ *k*+ 1.
   End while
Step 3. Algorithm termination. Output the optimal solution y* and the maximal market share $Z_{R}^{*}$.

In (REA), we embed (GCKP-A) to solve the inner-layer problem and use the 2-opt strategy to update the ranking score. Because (GCKP-A) is much faster than using other linear programming algorithms to solve the inner-layer problem, it can improve the computational efficiency of (REA). The 2-opt strategy can make the ranking results more comparable, so as to obtain a better combination of facility locations.

## 6 Computational experiments

### 6.1 Results for benchmark problems

By comparing the performance of our algorithm (REA) with the algorithm (RDOA-D) [16] on 40 benchmark problems (pmed1∼pmed40) [32], this subsection demonstrates the effectiveness of (REA). Note that computational experiments in [16] prove that the algorithm (RDOA-D) is superior to the algorithm (RDOA) and the genetic algorithm (GA) for all parameters of the tested problem, so we only compare the performance of algorithm (REA) and algorithm (RDOA-D). The number of nodes in these benchmark instances ranges from 100 to 900. The distance *d*_*ij*_ between the pair of points *i* and *j* is the Euclidean distance. All experiments are performed in a MATLAB environment on a laptop computer with Intel(R) Core(TM) i7–1065G7 @1.30 GHz CPU, and 16.00 GB of RAM.

First, for generalized continuous knapsack problems of different scales, we compare the efficiency of the algorithm (GCKP-A, A1 in Table 1) with the algorithm used for linear programming algorithm in Gurobi 9.1.1 (Linear-A, A2 in Table 1). For each example, the parameters *p*_*j*_ and *w*_*j*_ are randomly generated from intervals [−100, 100] and [10, 100], respectively. The optimal solutions obtained by the two algorithms are the same, and the CPU times used are shown in Table 1. It can be seen from Table 1 that the average CPU time used by (Linear-A) is more than 640 times that of (GCKP-A), so the calculation efficiency of (GCKP-A) is significantly higher than (Linear-A) of Gurobi. Therefore, using (GCKP-A) in (REA) instead of linear algorithm of Gurobi can greatly improve the efficiency of the algorithm (REA).

**Table 1:** Comparison of algorithm efficiency for solving (GCKP) (time unit: Second).

	*n* = 100	*n* = 200	*n* = 300	*n* = 400	*n* = 500	*n* = 600	*n* = 700	*n* = 800	*n* = 900	*n* = 1000	Mean
A1	0.0003	0.0003	0.0004	0.0002	0.0002	0.0002	0.0002	0.0004	0.0005	0.0005	0.00032
A2	0.1762	0.1899	0.1894	0.1859	0.1826	0.1925	0.1809	0.1857	0.2979	0.2896	0.2071

Next, we test the optimality of the algorithm (REA) and compare the performance of Simulated Annealing Algorithm (SA). For the first 5 benchmark examples (pmed1∼pmed5), we compare the results obtained by (REA) with the exact results (Market Share (MS)) obtained by solving the linearized model (LM). In each example, suppose that the first 10 locations are occupied by existing facilities, and the next |*J*_*N*_| locations are candidate facility locations for the new entrant. The quality *Q*_*j*_ of facility *j* is randomly generated as an integer number in [10, 100]. The customer demands of node *i* set to be *w*_*i*_ = 100/*i* and the fixed cost *f*_*j*_ of opening a new facility at candidate node *j* randomly generated as an integer number in [200, 500]. Other parameters are as follows: *R*_*c*_ = 60, *R*_*q*_ = 100, *δ* = 80, *γ*_1_ = 0.4, *γ*_2_ = 0.6, *G* = 2000. For each example, (REA) and (SA) were run 20 times, and the results are listed in Table 2. The Gap is defined as follows: $Gap=\frac{MS-Mean}{MS}\times 100\%$. From Table 2, we can find that the maximum gap between the average market share obtained from the algorithm (REA) and the exact result is no more than 0.35%. This verifies the optimality of the proposed algorithm (REA). On the other hand, the Gap of the Simulated Annealing Algorithm is larger than the Gap of (REA) for almost every example. Therefore, we can conclude that the performance of (REA) is better than (SA).

**Table 2:** Optimality of the algorithm (REA).

		Exact	(REA)				(SA)			
No.	|*J*_*N*_|	MS	Max.	Min.	Mean	Gap	Max.	Min.	Mean	Gap
#1	30	86.0944	86.0944	86.0944	86.0944	0%	86.0944	85.0634	85.8367	0.3%
	40	100.1909	100.1909	100.1909	100.1909	0%	100.1909	97.3497	99.8464	0.34%
	50	101.5025	101.5025	101.5025	101.5025	0%	101.5025	96.1879	99.8296	1.65%
#2	30	102.9141	102.9141	102.9141	102.9141	0%	102.9141	102.8386	102.9028	0.01%
	40	127.7107	127.7107	127.7107	127.7107	0%	127.7107	124.7100	126.6604	0.82%
	50	129.9127	129.9126	127.2366	129.4630	0.35%	129.9126	124.7341	129.6025	0.24%
#3	30	88.6559	88.6559	88.6559	88.6559	0%	88.6559	85.9735	88.3029	0.40%
	40	111.1247	111.1242	110.1094	111.0734	0.05%	111.1242	106.5701	110.8965	0.21%
	50	101.6469	101.6469	100.2887	101.5790	0.07%	101.6469	94.1251	99.8916	1.73%
#4	30	118.1794	118.1794	118.0909	118.1219	0.05%	118.1794	118.0105	118.0612	0.10%
	40	122.4177	122.4177	122.4177	122.4177	0%	122.4177	120.7804	122.2540	0.13%
	50	70.3602	70.3602	70.3602	70.3602	0%	70.3602	63.7387	69.7246	0.90%
#5	30	80.3325	80.3325	80.3325	80.3325	0%	80.3325	79.5055	80.2085	0.15%
	40	104.0804	104.0804	104.0804	104.0804	0%	104.0804	99.9589	103.8743	0.20%
	50	82.1296	82.1296	80.8194	82.0641	0.08%	82.1296	77.8385	81.7646	0.44%

Finally, we compared the performance of the algorithm (REA) with the algorithm (RDOA-D) using 40 benchmark instances. Similarly, assuming that in each example, the first 10 locations are occupied by existing facilities, the next |*J*_*N*_| locations are candidate facility locations for the new entrant. Except for the parameters given in Table 3, all other parameters are the same as those used in Table 2. We performed 20 calculations for each instance, and the mean and standard deviation of the market shares obtained are shown in Table 3. The larger mean and smaller standard deviation of the two algorithms are shown in bold.

**Table 3:** Performance comparison of (REA) and (RDOA-D).

〈#:*G*, |*I*|, |*J*_*N*_|〉	(REA)		(RDOA-D)		〈#:*G*, |*I*|, |*J*_*N*_|〉	(REA)		(RDOA-D)	
	Mean	Std.	Mean	Std.		Mean	Std.	Mean	Std.
1:2000,100,60	**93.966**	**0.185**	93.391	0.676	21:3000,500,60	**231.714**	**0.231**	229.980	1.769
2:2000,100,60	**118.544**	0.119	118.513	**0.077**	22:3000,500,60	**303.405**	**0.131**	303.088	0.416
3:2000,100,60	104.674	1.304	**104.845**	**1.180**	23:3000,500,60	**330.032**	**0.000**	329.904	0.299
4:2000,100,60	**104.694**	**0.178**	103.128	2.418	24:3000,500,60	**275.718**	**0.000**	275.592	0.163
5:2000,100,60	**144.267**	**2.530**	141.658	2.801	25:3000,500,60	**334.615**	**0.537**	333.547	0.816
6:2000,200,80	**151.319**	**0.520**	150.159	0.737	26:3000,600,80	**276.799**	**0.000**	276.541	0.557
7:2000,200,80	179.535	1.470	**180.449**	**0.783**	27:3000,600,80	**332.570**	**0.000**	331.917	0.630
8:2000,200,80	234.200	0.000	234.200	0.000	28:3000,600,80	**318.632**	**0.000**	318.462	0.225
9:2000,200,80	**153.915**	**0.906**	150.949	1.941	29:3000,600,80	**301.130**	**0.360**	300.459	0.443
10:2000,200,80	**225.614**	**0.000**	224.554	1.367	30:3000,600,80	**315.423**	**0.798**	314.436	1.294
11:2500,300,80	**239.610**	**0.529**	237.713	1.160	31:3500,700,80	**295.503**	**0.000**	294.107	1.279
12:2500,300,80	**247.627**	**0.000**	247.512	0.182	32:3500,700,80	**305.194**	**0.161**	303.251	0.912
13:2500,300,80	**206.564**	**0.105**	206.491	0.172	33:3500,700,80	**320.562**	**0.000**	319.979	0.506
14:2500,300,80	**252.478**	**0.107**	252.074	0.719	34:3500,700,80	**383.017**	**0.107**	382.329	0.500
15:2500,300,80	**232.803**	**0.638**	232.243	1.182	35:3500,800,80	**348.047**	**0.000**	347.483	0.499
16:2500,400,100	288.557	0.134	**288.621**	**0.000**	36:3500,800,100	**339.951**	**0.584**	336.575	1.323
17:2500,400,100	**230.678**	1.144	229.300	**1.023**	37:3500,800,100	**349.700**	**0.279**	347.553	1.144
18:2500,400,100	**302.579**	**0.570**	301.718	0.579	38:3500,900,100	**337.952**	0.874	335.746	**0.788**
19:2500,400,100	**314.042**	**0.000**	311.155	2.262	39:3500,900,100	**334.147**	**0.278**	332.247	0.892
20:2500,400,100	**306.630**	**1.137**	302.049	1.548	40:3500,900,100	**389.986**	0.681	387.673	**0.420**

It can be clearly seen from Table 3 that the algorithm (REA) is superior to the algorithm RDOA-D in both the mean and standard deviation of the market share.

### 6.2 A quasi-real example

In order to investigate the effects of different parameters in the presented competitive facility location model. This subsection discusses a quasi-real example based on the 49-nodes data set described in Daskin [33]. This data set consists of the capitals of the continental United States and Washington, DC.

The customer demands *d*_*i*_ is proportional to the population of the state (*i*) in 1990. The opening cost *f*_*j*_ of the potential facility location *j* ∈ *J*_*N*_ also comes from Daskin’s data set. In this example, we generated a set of random numbers within [10, 120] to represent the facility quality *Q*_*j*_ at each point *j* ∈ *J*. Now, there are 5 facilities located in the states of California, Kentucky, New York, Texas, and Wyoming, respectively. The qualities of these 5 facilities are 106, 98, 107, 45, and 106 respectively. To avoid being too close, we multiply the Euclidean distance between any two points by 10, and then round it to *d*_*ij*_. Other parameters are defined as follows: *G* = 200000, *δ* = 100, *R*_*c*_ = 40, *R*_*q*_ = 80, *γ*_1_ = 0.4, *γ*_2_ = 0.6.

The best solution to the problem of the new entrant’s competitive facility location is to open three facilities in Georgia, Indiana, and Missouri. The newly-entered company’s market share from its three facilities is 7821.1. The locations of the new facilities and customers served are shown in Table 4.

**Table 4:** Location scheme of the new entrant (*γ*_1_ = 0.4, *γ*_2_ = 0.6).

Location	Demand type	Customers Served
Georgia	Convenience	Alabama, Florida, Georgia, South Carolina, Tennessee
Indiana	Quality/Convenience	Alabama, Arkansas, Georgia, Illinois, Indiana, Iowa, Kentucky, Michigan, Missouri, Ohio, South Carolina, Tennessee, West Virginia, Wisconsia
Missouri	Convenience	Arkansas, Illinois, Iowa, Kansas, Missouri

Let *γ*_1_ change from 0.1 to 0.9 with a step size of 0.1 to test the impact of *γ*_1_, *γ*_2_ on the location scheme while maintaining *γ*_2_ > *γ*_1_. We found that when *γ*_1_ ≤ 0.8, no matter what value *γ*_2_ takes in [*γ*_1_, 1], the location scheme is exactly the same as that shown in Table 4. Only when *γ*_1_ ≥ 0.9, the location scheme will be different. In this case, the new company will build three facilities in Georgia, Indiana, and Pennsylvania. Table 5 shows the location scheme when *γ*_1_ = 0.9, *γ*_2_ = 1. The market share captured by the new entering firm is 8159.2, which is larger than the previous market share 7821.1. This reflects that the reduced degree of uncertainty can bring more market share to the new entrant.

**Table 5:** Location scheme of the new entrant (*γ*_1_ = 0.9, *γ*_2_ = 1).

Location	Demand type	Customers Served
Georgia	Convenience	Alabama, Florida, Georgia, South Carolina, Tennessee
Indiana	Quality/Convenience	Alabama, Arkansas, Georgia, Illinois, Indiana, Iowa, Kentucky, Michigan, Missouri, Ohio, South Carolina, Tennessee, West Virginia, Wisconsia
Pennsylvania	Convenience	Delaware, Maryland, New Jersey, New York, Pennsylvania, Virginia

In addition to *γ*_1_, *γ*_2_, there are other important parameters in the model such as *G*, *R*_*q*_, *R*_*c*_, *δ*, etc. Now we analyze the influence of these parameters on the market share of the newly entered company.

**Fig 4:**
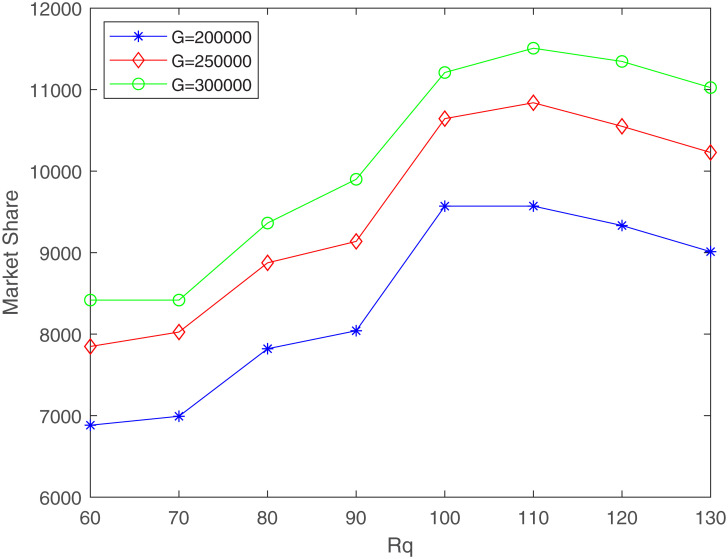
The relationship between market share and *R*_*q*_ (*R*_*c*_ = 40, *δ* = 100, *γ*_1_ = 0.4, *γ*_2_ = 0.6).

Firstly, we analyze the impact of *R*_*q*_ on market share. Set the budget *G* to 200000, 250000, and 300000 respectively. For each value of *G*, let *R*_*q*_ change from 60 to 130 with a step of 10, we can get a curve representing the change of market share with *R*_*q*_. The three curves with different budgets are shown in Fig 4.

It can be clearly seen from Fig 4 that for different budgets, with the increase of *R*_*q*_, the market share first increases and then decreases. Due to the increase in *R*_*q*_, the number of demand points that can be met by high-quality facilities increases, so the market share first increases with the increase in *R*_*q*_. With the further increase of *R*_*q*_, the coverage of existing high-quality facilities has also increased, leading to intensified competition among facilities, so the available market share of the newly entered firm has begun to decline.

**Fig 5:**
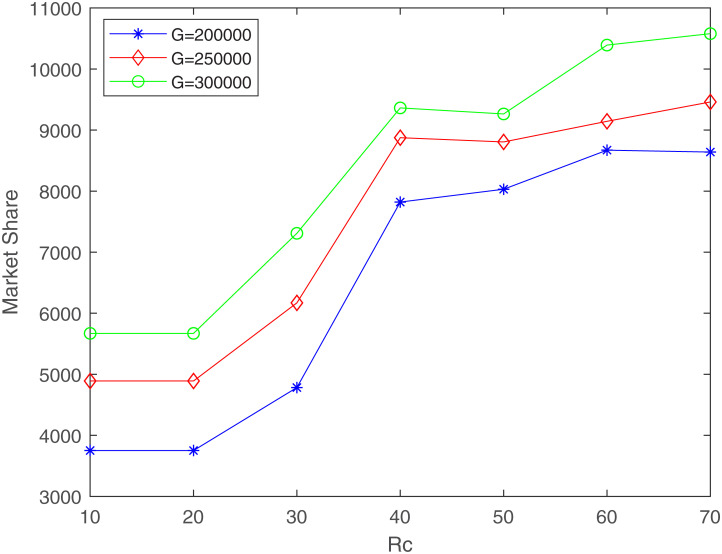
The relationship between market share and *R*_*c*_ (*R*_*q*_ = 80, *δ* = 100, *γ*_1_ = 0.4, *γ*_2_ = 0.6).

Secondly, we analyze the impact of *R*_*c*_ on market share. We still set the budget *G* to 200000, 250000, and 300000 respectively. Then change *R*_*c*_ from 10 to 70 in step size of 10. The relationship curves between market share and *R*_*c*_ are shown in Fig 5.

It can be seen from Fig 5 that the market share increases with the increase of *R*_*c*_. When *R*_*c*_ changes from 10 to 20, the demand points covered by low-quality facilities are unchanged, so the market share remains the same. When *R*_*c*_ changes from 20 to 40, the demand points covered by low-quality facilities increase, so the market share increases significantly. When *R*_*c*_ further increases, competition among low-quality facilities begins to emerge, and therefore, the growth of market share slows down.

**Fig 6:**
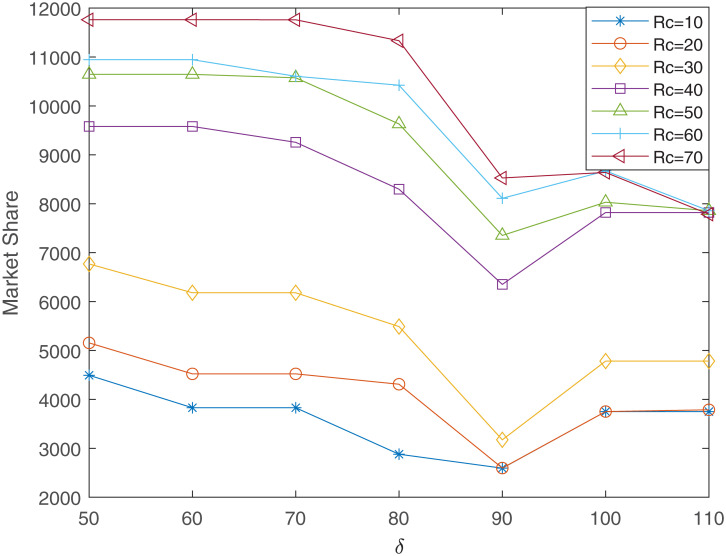
The relationship between market share and *δ* (*R*_*q*_ = 80, *γ*_1_ = 0.4, *γ*_2_ = 0.6).

Thirdly, we analyze the impact of *δ* on market share. Let *R*_*q*_ = 80 and *δ* changes from 50 to 110 in step size of 10, for 7 different values of *R*_*c*_, the relationship curves between market share and *δ* are shown in Fig 6.

It can be seen from Fig 6 that when *δ* changes from 50 to 90, the market share decreases as *δ* increases. This is because with the increase of *δ*, the number of facilities that meet the quality requirements higher than *δ* gradually decreases. When *δ* changes from 90 to 100, the market share increases as *δ* increases. This is due to the reduction in the number of existing facilities that meet the quality higher than *δ*, resulting in less competition among high-quality facilities. When *δ* changes from 100 to 110, the market share does not increase with the increase of *δ*. This is because when the *δ* further increases, the number of facilities that meet the quality requirements decreases significantly, so the market share that the new entrant can obtain remains unchanged or slightly decreases. For any value of *R*_*c*_, when *δ* = 90, the market share reaches the minimum value. In order to study the reason in-depth, we take *R*_*c*_ = 40 as an example, and the locations of *δ* = 80, 90, 100 are listed in Table 6.

**Table 6:** The location scheme for different *δ* (*R*_*c*_ = 40, *R*_*q*_ = 80, *γ*_1_ = 0.4, *γ*_2_ = 0.6).

*δ* = 80			*δ* = 90			*δ* = 100		
Locations	*Q*_*j*_	MS	Locations	*Q*_*j*_	MS	Locations	*Q*_*j*_	MS
Arkansas	91	8297.5	Arkansas	91	6351.1	Georgia	36	7821.1
Indiana	111		Indiana	111		Indiana	111	
Virginia	82		Kansas	67		Missouri	24	

It can be seen from Table 6 that when *δ* increases, the number of high-quality facilities (*Q*_*j*_ underlined in Table 4) in the selected facilities gradually decreases. Denote the solution when *δ* = 80 as $y_{\delta =80}^{*}$, then the market share corresponding to this solution when *δ* = 90 is 4887. Similarly, denote the solution when *δ* = 100 as $y_{\delta =100}^{*}$, then the market share corresponding to this solution when *δ* = 90 is 5496.8.

**Fig 7:**
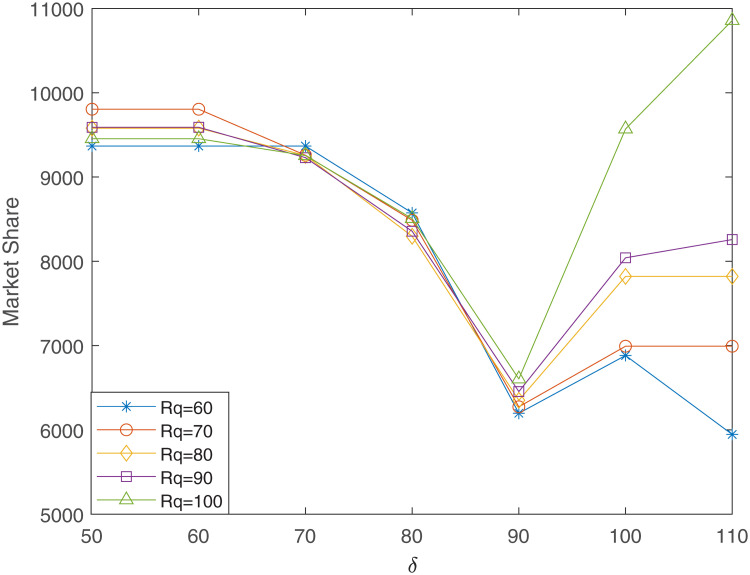
The relationship between market share and *δ* (*R*_*c*_ = 40, *γ*_1_ = 0.4, *γ*_2_ = 0.6).

Similarly, let *R*_*c*_ = 40 and *δ* changes from 50 to 110 in step size of 10, for different values of *R*_*q*_, the relationship curves between market share and *δ* are shown in Fig 7.

It can be seen in Fig 7 that when *δ* ≤ 90, the impact of *R*_*q*_ on the market share is not obvious. But when *δ* ≥ 100, there is a clear positive correlation between market share and *R*_*q*_. This is because as *δ* increases, the number of existing facilities meeting high-quality requirements decreases. Therefore, for the new entrant, the market share brought about by choosing high-quality facilities will increase with the increase of *R*_*q*_.

Finally, we analyzed the impact of the budget *G* on the location of the new entrant’s facilities, and the results are listed in Table 7. The last raw in Table 7 indicates the market share of the new entrant.

**Table 7:** The location scheme for different *G* (*R*_*c*_ = 40, *R*_*q*_ = 80, *δ* = 100, *γ*_1_ = 0.4, *γ*_2_ = 0.6).

*G* = 100000		*G* = 150000		*G* = 200000		*G* = 250000		*G* = 300000	
Locations	*Q*_*j*_	Locations	*Q*_*j*_	Locations	*Q*_*j*_	Locations	*Q*_*j*_	Locations	*Q*_*j*_
Indiana	111	Georgia	36	Georgia	36	Georgia	36	Alabama	31
		Indiana	111	Indiana	111	Indiana	111	Indiana	111
				Missouri	24	Iowa	16	Iowa	16
						South Dakota	114	Ohio	51
								South Dakota	114
								West Virginia	52
4321.0		6224.7		7821.1		8875.0		9363.0	

It can be seen from Table 7 that the market share of the new entrant increases as the budget increases. An interesting phenomenon is that for a multi-stage budget situation, it is also possible to obtain the overall optimal solution. For example, suppose that the first stage budget is 150000, and the second stage budget is 100000. We can choose Georgia and Indiana in the first stage, and then choose Iowa and South Dakota in the second stage. This is exactly the optimal location scheme under the total budget of 250000.

## 7 Conclusion

Observing that many customers often have both convenience and quality demands, we propose a new customer choice rule to describe this behavior. In fact, if the quality threshold *δ* is set to infinity, the customer choice rule based on convenience range proposed by Qi et al. [23] can be regarded as a special case of the rule in this paper. By considering the uncertainty of the proportion of convenience-type demands in the total demands, we proposed a two-layer robust model to study the competitive facility location problem.

The initial two-layer robust model is linearized as a mixed binary linear programming problem. This allows us to solve exactly medium size problems. For large size problems, we first prove a theorem for the optimal solution of the generalized continuous Knapsack problem. Then we propose an exact algorithm (GCKP-A) to solve the inner-layer model. By imbedding (GCKP-A) and 2-opt strategy into the framework of the improved ranking-based algorithm, we propose the heuristic algorithm (REA) for large size problems. On the one hand, the solution of the proposed algorithm (REA) is compared with the exact solution for different benchmark examples, which verifies the optimality of the proposed algorithm. On the other hand, the comparison of the performance of the algorithm (REA) and the algorithm (RDOA-D) reflects the superiority of the algorithm (REA).

A quasi-real example is used to illustrate the impact of different parameters on the market share of the new entrant. Four main conclusions are obtained through sensitivity analysis: (1) Only when the proportion of convenience-type demands is high, will it affect the robust location scheme; (2) The market share of the new entrant increases first and then decreases with the increase of *R*_*q*_; (3) The market share of the new entrant increases with the increase of *R*_*c*_; (4) The market share of the new entrant decreases first and then does not decrease with the increase of *δ*.

In this article, we only studied the competitive facility location problem in a static environment. Based on the classification of these two types of demands, we should consider the reaction of the competitors in the location of competitive facilities. The leader-follower competitive facility location problem based on the newly proposed customer choice rule deserves to be studied in future work.

## Supporting information

S1 File The data set of the 49-nodes example in subsection 6.2 is provided as S1 File. The first two columns of the data are latitudes and longitudes of the capitals of the continental United States plus Washington, DC. The third column denotes the existing facilities. The fourth column is the demand *d*_*i*_. The fifth column denotes the quality of facilities. The sixth column represents the cost of opening a facility at position *j*. The last column is the name of the states.
(XLS)
